# The effect of mesenchymal stem cell-conditioned medium gel on burn wound healing in rat

**DOI:** 10.14202/vetworld.2022.841-847

**Published:** 2022-04-07

**Authors:** Dian Ratih Laksmitawati, Siti Umrah Noor, Yati Sumiyati, Adrian Hartanto, Wahyu Widowati, Diah Kartika Pratami

**Affiliations:** 1 Laboratory of Biochemistry, Faculty of Pharmacy, Universitas Pancasila, Jakarta, 12640, Indonesia; 2 Laboratory of Pharmaceutical Technology, Faculty of Pharmacy, Universitas Pancasila, Jakarta, 12640, Indonesia; 3 Medical Research Center, Faculty of Medicine, Maranatha University, Bandung, West Java, 40164, Indonesia; 4 Laboratory of Pharmacognosy and Phytochemistry, Faculty of Pharmacy, Pancasila University, Jakarta, 12640, Indonesia

**Keywords:** conditioned medium, mesenchymal stem cell, third-degree burn

## Abstract

**Background and Aim:**

Stem cells are cells that can proliferate to form a new tissue, leading to its use in regenerative therapy. Stem cells will secrete biological factors, such as growth factors, cytokines, and other proteins to their surroundings and culture medium/conditioned medium (CM), altering tissue physiology. These factors can help wound healing, but their effect on third-degree burns is poorly understood. This research aimed to study the activity of mesenchymal stem cell-conditioned medium gel in healing and repairing third-degree burns on rats skin.

**Materials and Methods:**

Twenty-four Sprague–Dawley rats with burn wounds on the dorsal area were divided into four groups; the first group was treated with CM gel, with a concentration equivalent to 0.05% protein, the second group was treated with a placebo gel, the third group with silver sulfadiazine (SSD) cream (SSD-Burnazin contain 10 mg/g SSD), and the fourth group was not given any treatment, for 21 days, and on the final day, the rats were sacrificed, and the skins were taken. All topical treatments completely cover the wound area.

**Results:**

Wound healing process indicators observed include wound diameter, scabs’ formation, blister formation, and hair growth every day. The skins taken were processed with hematoxylin-eosin and Masson’s trichrome staining. The indicators studied include neutrophil infiltration, mononuclear cell infiltration, neovascularization, collagen area, and re-epithelization ratio.

**Conclusion:**

CM shows better wound healing than other groups and faster hair growth.

## Introduction

The skin is an organ that protects the body from exogenous substances. There are numerous factors that can damage the body, resulting in permanent damage and even death. One of them is a burn wound because a burn wound can destroy all skin layers from the epidermis up to the hypodermis and other tissues, such as blood vessels, neurons, tendons, and bone, which can increase the chance of severe infection [1]. Histologically, third-degree burns (full-thickness burn) damage all parts of the dermis and sometimes involve the subcutaneous adipose tissue. Without any operation, this type of burn wound can be recovered if wound contracture occurs or by skin grafting method [2].

Wound healing occurs in a few phases: vascular response, inflammation, proliferation, and tissue recovery, and ends with the remodeling phase [3]. Many cytokines and growth factors affect these phases [3,4]. Bad blood flows, diabetes mellitus, and pressure ulcer are the primary causes of impaired wound healing. Other factors, including nutrition, immune system, age, stress, and other comorbid factors can also cause impaired wound healing [5].

A stem cell is a cell that has not differentiated, having a unique ability to differentiate into another cell type. This ability makes the stem cell responsible for tissue recovery [6,7]. A stem cell can be attributed to three key mechanisms of action and one of them is differentiation into multiple cell types, which locally engrafts and induces the restoration of function by augmenting or replacing damaged tissues [8]. The other stem cell mechanism of action is the secretion of bioactive factors, which may affect both systemic and local physiological processes [8]. Some researchers have shown that during stem cell growths, organic molecules such as growth factors, cytokines, and other proteins are secreted into the culture medium [7-9]. This culture medium is called a conditioned medium (CM).

CM usage in tissue regeneration has been performed many times as *in vitro* or *in vivo* studies. *In vitro* study showed that mesenchymal stem cell-CM (MSC-CM) can increase keratinocytes and fibroblast migration, and help in the extracellular matrix formation [10]. *In vivo* studies showed that the use of CM on wound resulted in better wound healing on parameters, such as re-epithelization, granulocyte tissue formation, vascularization, and wound margins distance [11-13].

The use of CM on third-degree burns has not been fully understood; thus, this research aimed to study the activity of mesenchymal stem cell-conditioned medium gel in healing and repairing third-degree burns on rat skin. CM was developed into gel form using hydroxypropyl methylcellulose (HPMC) as the gel base for making the CM application easier.

## Materials and Methods

### Ethical approval

The Faculty of Medicine, Universitas Indonesia, and Cipto Mangunkusumo Hospital Ethics Committee approved the ethical approval evaluation (0966/UN2.F1/ETIK/2018). ARRIVE guidelines were followed for this study.

### Study period and location

The study was conducted from May to December 2018 at the Faculty of Pharmacy, Pancasila University, Jakarta, Indonesia. Histology preparation was conducted at the Pusat Studi Satwa Primata laboratory, IPB University, Bogor, West Java, Indonesia.

### CM preparation

The CM was collected from adipose-derived stem cell culture medium passages 3-14 from Biomolecular Aretha Medika Utama, Bandung, Indonesia. CM was kept under −20°C before usage. The culture medium used for the stem cell was Minimum Essential Medium Eagle Alpha Modification (MEM-α) and 10% fresh frozen plasma (FFP) in normoxia condition. The cultured cells had fulfilled the phenotype characteristics, which include CD44, CD73, CD90, and CD105 positive, and CD11b, CD19, CD34, CD45, and HLA-DR negative [14].

### CM gel formulation

Before the CM was developed into a gel form, the total protein in CM was analyzed using Bradford protein assay. After the total protein in CM was known, it was developed into a gel with the following formula using the method described by Mappa *et al*. [15], and Sutrisno *et a*l. [16], with slight modification (Table-1). The placebo gel was made using a complete medium (MEM-α+10% FFP) to substitute the CM in the formula for the control. The final protein concentration was determined using the methods described by Timmers *et al*. [17] experiment.

**Table 1:** Formulation of CM [15,16].

Ingredients	Quantity
CM	Equal 0.05% protein
HPMC	2%
Glydant^®^ Plus Liquid	0.3%
Propylene glycol	10%
Demineralized water	Ad 100%

CM=Conditioned medium, HPMC=Hydroxypropyl methylcellulose

### Animal model

This study was conducted using 24 male albino Sprague–Dawley rats, of 12-14 weeks, weighing 200-250 g, obtained from Bogor Agricultural University (IPB). All rats underwent acclimatization for 1 week, maintained in cages of 90×60×60 cm, at 24±2°C on a 12 h dark/light cycle, and were given feed and water *ad libitum*. Animals were excluded if the rat looked sick or died, or decreased weight loss during the acclimatization period by more than 10%.

### Burn wound induction and treatment

The rats were randomly grouped into four groups, with each group consisting of six rats based on Federer sample size formulation. All groups except the fourth group were burn induced. The burn wound in the first rat group was treated with CM gel (with a concentration equivalent to 0.05% protein), the second group was treated with a placebo gel, the third group was treated with silver sulfadiazine (SSD) cream (SSD-Burnazin contain 10 mg/g SSD), and the fourth group was not administered any treatment. The burn wound induction was performed using methods described by Fahimi *et al*. [18] and Mehrvarz *et al*. [19]. One day before burn induction, the rat’s dorsum hair was shaved and left for one night before the burn induction. On the next day, the rats were intraperitoneally anesthetized using 60 mg/kg ketamine, before the burn wound was induced. The burn wounds were induced using round iron of diameter 18 mm, which had been heated using Bunsen for 3 min. The iron was contacted to the dorsal area or the back of the rat for 10 s without force.

One burn wound was induced for each rat and treated with CM gel using the method described by Sutrisno *et al*. [16]. There were four groups, and each group consisted of six rats. The first group was treated with CM gel (with a concentration equivalent to 0.05% protein), the second group was treated with a placebo gel, the third group was treated with SSD cream (SSD-Burnazin contain 10 mg/g SSD), and the fourth group was not administered any treatment. The wounds were treated for 21 days by applying the gel or cream once daily until it covered all wound surfaces.

### Wound healing scoring

Wound anatomy, such as wound diameter, was observed daily by finding the average vertical and horizontal length of the wound, hair growth, and scab formation. On the 21^st^ day, all rats were sacrificed. The skin was collected and fixed using 10% neutral buffered formalin and further processed and stained using hematoxylin-eosin staining, followed by Masson’s trichrome staining. Observed parameters include neutrophil infiltration, mononuclear cell infiltration, neovascularization, collagen thickness, and re-epithelization ratio using a light microscope (Olympus BX 31, Olympus Optical Co., Ltd., Tokyo, Japan) equipped with a CCD10 USB Camera with 40× and 400×. The wound healing scoring was made using the method described by Sutrisno *et al*. [16] and Mehrvarz *et al*. [19]. Histology preparation and scoring were blindly conducted by experts from the Pusat Studi Satwa Primata laboratory, IPB University, Bogor, West Java, Indonesia.

### Statistical analysis

Hair growth and scab formation data were analyzed descriptively, while wound diameter and histology data were analyzed statistically. Wound diameter was analyzed using the one-way analysis of variance (ANOVA) test, while histology data were analyzed using the Kruskal–Wallis tests.

## Results

### Burn degree identification

**Figure-1:**
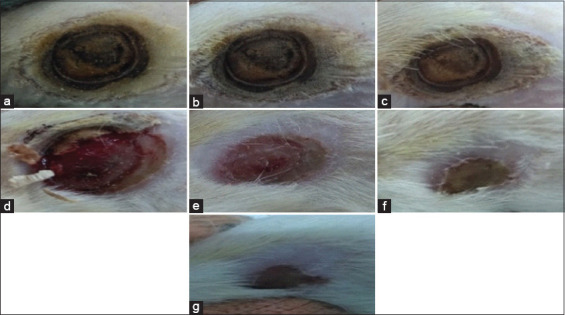
Clinical progression of third-degree burn wound healing on Sprague–Dawley rat; (a) wound on day 0 a moment after induction; (b) wound on day 4, there was no significant difference observed; (c) wound on day 8, there was no significant difference observed but scab was starting to be formed; (d) wound on day 12, scab was nearly off; (e) wound on day 15, redness was beginning to fade and the wound diameter was shorter than before; (f) wound on day 18, wound became dry and the diameter was reduced further; (g) wound on day 21, wound diameter was reduced further but the wound healing was not completed yet.

**Figure-2:**
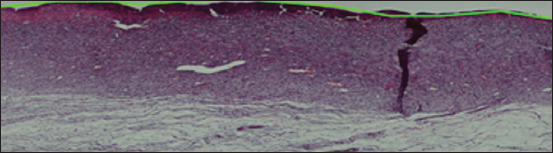
Skin tissue (hematoxylin and eosin staining); damage had reached all the dermis and the tissue below the dermis; there were no hair follicles or oil glands found on the dermis layer.

After the rats were induced with burn wound, the wounds appeared brown and had a leathery pattern at the center, a whitish area at the outer ring of the center, and a reddish line at the outer of the whitish area. The brown area was expected to be the necrosis zone, the white one as the stasis zone, and the red area as the hyperemia zone (Figure-1). After that, the skins were stained using H and E staining. The damage shown in Figure-2 had reached the subcutaneous layer; therefore, the wounds were identified as third-degree burns [20].

### Wound anatomy parameter

**Figure-3:**
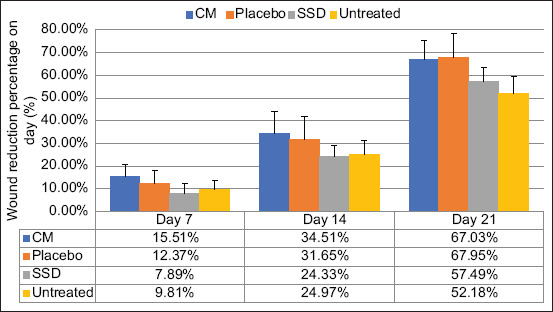
Average of wound diameter reduction on days 7, 14, and 21. Data are given in average+SD, n=6, CM=Conditioned medium, SSD=Silver sulfadiazine.

The average wound diameter reduction on days 0, 7, 14, and 21 is shown in Figure-3. Using the ANOVA test, the wound reduction percentage showed a significant difference (p<0.05) on day 21. The statistical test *post hoc* Tukey showed a significant difference between wound reduction of CM and placebo group with the untreated group (Table-2).

**Table 2:** Wound percentage reducement on day 21.

Group	Wound reduction percentage on day 21 (%)
CM	67.03±8.38^a^
Placebo	67.95±10.37^a^
SSD	57.49±5.85^a,b^
Untreated	52.18±7.31^b^

Data were given in average+SD, n=6, CM=Conditioned medium, SSD=Silver sulfadiazine

Besides wound diameter, the other observed parameters include scab formation, blister formation, and hair growth, but during this study, no blister was formed. During this study, scab formation was assumed when there was a hard layer on the wound, and that layer seemed off on the outer ring. Scabs were formed on all groups on a day nearly similar to each other (Table-3). During this study, hair growth was assumed when there was hair on the wound. The CM group showed faster hair growth than other groups (Table-4).

**Table 3:** Days of scab formation and the scab off from the wound.

Group	Scab formed (day)	Scab off (day)
CM	5-6	14-16
Placebo	5-8	13-17
SSD	5-8	16-18
Untreated	5-8	13-15

CM=Conditioned medium, SSD=Silver sulfadiazine

**Table 4:** Days hair started to grow.

Group	Hair started to grow (day)
CM	3-8
Placebo	10-13
SSD	12-17
Untreated	13-19

CM=Conditioned medium, SSD=Silver sulfadiazine

### Wound histology parameter

**Figure-4:**
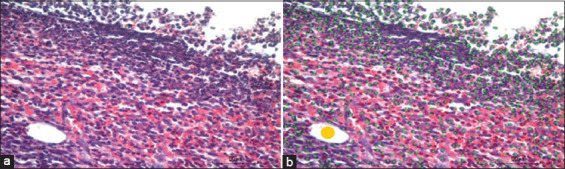
Wound histology on day 21 (hematoxylin and eosin staining, 400×); (a) original photo; (b) the counting, green dots show neutrophil, yellow dots show neovascularization, and red dots show mononuclear cell.

**Figure-5:**
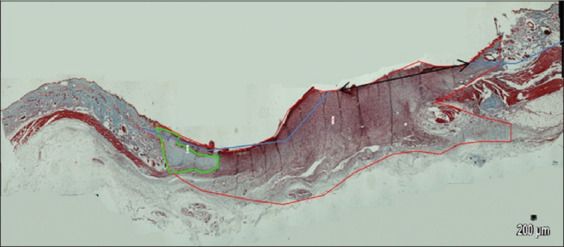
Wound histology on day 21 (Masson’s trichrome staining, 40×, pictures were combined using software PTGui (Pro software by New House Internet Service B.V; Rotterdam, the Netherlands); red line shows the wound area; green line shows collagen area; two-headed arrow shows the edges of re-epithelization area.

Histology parameters were observed by counting and observing the cell morphology. Data were collected from 10 different visual ranges done in a zigzag pattern to avoid the same cell being counted twice. An example of how to count the cell is shown in Figures-4 and 5, while the results are shown in Table-5.

**Table 5:** Histology parameters day 21.

Group	Neutrophil infiltration	Neovascularization	Mononuclear cell infiltration	Collagen area (mm^2^)	Re-epithelization ratio
CM	204.68±227.02	15.96±13.25	60.2±76.83	3.26±2.16^ab^	0.69±0.16
Placebo	150.06±165.66	18.76±16.52	68.78±86.54	6.15±1.65^a^	0.56±0.14
SSD	183.44±208.29	28.18±18.64	49.5±67.24	6.33±2.44^a^	0.52±0.06
Untreated	137.62±185.44	21.34±13.49	47.5±79.63	2.53±2.19^b^	0.71±0.18

Data were given in average±SD, n=6, CM=Conditioned medium, SSD=Silver sulfadiazine, SD=Standard deviation

The statistical result of Kruskal–Wallis test showed an insignificant difference for neutrophil infiltration, neovascularization, mononuclear cell infiltration, and the re-epithelization ratio (p>0.05), but there is a significant difference for collagen area parameter (p<0.05). The test is followed with *post hoc* Mann–Whitney U-test, which showed a significant difference between the placebo and SSD group with the untreated group (Table-5).

## Discussion

On the formula, HPMC was used as gel basis, propylene glycol as humectant and penetration enhancer, Glydant^®^ Plus Liquid (1,3-dimethylol-5,5-dimethylhydantoin and 3-iodo-2-propynyl butyl carbamate; Lonzagroup, Basel, Switzerland) as the preservative, and demineralized water as the base. Gel form was chosen because the gel has a high water content; therefore, it seemed suitable for burn wound application to avoid skin dehydration [21]. HPMC is a hydrocolloid type base, suitable for burn wound application. Such base is permeable to water vapor but impermeable to bacteria. It can also stimulate granulation tissue formation [22,23].

This study shows a significant difference in the wound reduction percentage between CM and placebo groups from the untreated group. This result shows that CM and MEM-α+FFP 10% have better wound healing activity than SSD cream. This phenomenon might be explained because CM and placebo consist of growth factors and other proteins, which affect wound healing phases [3,4], while SSD is an antibacterial agent that can delay wound re-epithelization [24]. *In vitro* study shows that MSC-CM can fasten fibroblast and keratinocyte migration which was suspected because of growth factors, cytokines, and other proteins in MSC-CM [10].

During the study, scabs were formed on all groups. Scab formation aims to close the wound so bacteria and debris from outside cannot enter and to keep humidity beneath the scab. However, the scab will slow the re-epithelization process because the collagen and fibrin layer can disturb epidermal cell migration from hair follicles and oil glands [22,23]. After the new epidermis layer beneath the scab is mature, the scab will pull off from the wound because the new tissue will push collagen, stretch the fibrin, or dissolve the collagen because of enzymes produced by epidermal cells and leukocytes [25]. This phenomenon can explain the big difference between wound reductions percentages on day 14 compared to day 21.

On the growth hair parameter, the CM group showed faster hair growth, followed by the placebo group. These data show a similar result with other studies on the use of CM on alopecia treatment in humans [26] and rats [27], suggesting that CM treatment can stimulate hair growth. Those studies suggest that growth factors in CM cause hair growth. The same reason possibly causes the result of this study because theoretically, CM consists of the highest number of growth factors in the samples used during this study followed by the placebo group since FFP consists of growth factors too though CM comprises a larger amount of growth factor.

Neutrophil and mononuclear cell counting results showed no significant difference, but descriptively, there are a larger number of these cells in the CM and placebo group than the SSD and untreated group. Inflammation cell infiltration occurs during the inflammation phase, and these cells will “eat” the bacteria and dead cells on the wound. It also produces cytokine that affects the following phases of wound healing [4]. According to this theory, it can be said that more infiltration occurring during the inflammation phase will result in better wound healing. This speculation is strengthened by the fact that the CM and placebo groups have the highest number of neutrophils and mononuclear cells than the other groups resulting in better wound healing.

The fewest neovascularization showed by the CM group can be caused when wound healing reaches the remodeling phase. The remodeling phase usually occurs on day 21 up to 1 year after the wound occurred, and during this phase, angiogenesis and blood flow will be reduced [4]. Theoretically, some growth factors can increase angiogenesis during the proliferation phase that occurred during day 3 up to day 10 [3,4]. Therefore, if the wound healing is still in the proliferation phase, CM and placebo groups should show the largest amount of neovascularization than the untreated and SSD groups.

CM shows the best re-epithelization ratio compared to placebo and SSD groups for the re-epithelization ratio data. Keratinocytes and stem cells from hair follicles or oil glands play an important role in the re-epithelization process. This result might be caused by growth factors and cytokines in CM, which are higher than FFP, resulting in better keratinocyte and stem cell migration, which was proved by another study on the use of MSC-CM, leading to better migration of fibroblast and keratinocyte [28]. The high ratio for the untreated group might be caused by the rat’s effort to keep the wound from infection because the tissue below epidermis wound healing is poor. This hypothesis needs to be tested further to be proven.

The collagen result showed that CM has fewer collagens than placebo and SSD groups. This might be caused by the CM mechanism of action, which is similar to one of the stem cells’ mechanism of action, which is the paracrine effect, and the use of stem cell can reduce collagen type I amount, leading to better scarring [29]. Another factor that might cause this phenomenon is that CM gel and CM itself have a higher pH (base) compared to MEM-α+FFP 10% because fibroblast produces collagen in an acid environment [30]. Rats and humans have similar tissue structure and physiology; therefore, the rat burn model and healing process data can provide useful information extrapolated to humans.

## Conclusion

The application of CM gel with a concentration equivalent to 0.05% protein using HPMC as the gel base shows better wound healing. The use of CM gel treated Sprague–Dawley rats with burn wounds on the dorsal shows faster hair growth compared to the placebo gel, SSD cream, and the non-treatment groups. CM is an active ingredient that promises to be used as a topical preparation with regeneration properties. As a topical preparation, standardization of CM production is required.

## Authors’ Contributions

DRL, SUN, WW, and YS: Arranged, designed, and supervised the study. AH: Carried out sampling, laboratory analysis, and wrote the first draft of the manuscript. DRL and WW: Analyzed the data. DRL, WW, and DKP: Contributed to the writing of the manuscript. DRL, SUN, YS, AH, WW, and DKP: Acquisition, analysis, and interpretation of data. DKP, AH, and DRL: Made critical revisions. All authors read and approved the final manuscript.
